# Motor-cognitive coupling is impaired in children with mild or severe forms of developmental coordination disorder

**DOI:** 10.3389/fnhum.2023.1252852

**Published:** 2023-10-24

**Authors:** Reza Abdollahipour, Ludvík Valtr, Kamila Banátová, Lucia Bizovská, Tomáš Klein, Zdeněk Svoboda, Bert Steenbergen, Peter Henry Wilson

**Affiliations:** ^1^Department of Natural Sciences in Kinanthropology, Faculty of Physical Culture, Palacký University Olomouc, Olomouc, Czechia; ^2^Department of Pedagogical and Educational Sciences, Behavioural Science Institute, Radboud University, Nijmegen, Netherlands; ^3^Healthy Brain and Mind Research Centre, School of Behavioural and Health Sciences, Faculty of Health Sciences, Australian Catholic University, Melbourne, VIC, Australia

**Keywords:** developmental coordination disorder (DCD), children, motor control, motor inhibition, goal-directed action

## Abstract

Children with developmental coordination disorder (DCD) show deficits in motor-cognitive coupling. However, it remains unclear whether such deficits depend on the severity of DCD. The aim of this study was to examine cognitive-motor coupling under different levels of inhibitory control in children with severe (s-DCD) or moderate DCD (m-DCD), compared with typically-developing children (TDC). The performance of 29 primary-school children aged 6–12 years with s-DCD (*M*age = 9.12 ± 1.56 years), 53 m-DCD (*M*age = 8.78 ± 1.67 years), and 201 TDC (*M*age = 9.20 ± 1.50 years) was compared on a double jump reaching task (DJRT) paradigm, presented on a large 42-inch touchscreen. The task display had a circular home-base, centred at the bottom of the display, and three target locations at radials of −20°, 0°, and 20°, 40 cm above the home-base circle. For the standard double-jump reaching task (DJRT), children moved their index finger from home-base circle to touch the target stimulus as fast as possible; 20% were jump trials where the target shifted left or right at lift-off. For the anti-jump reaching task (AJRT), 20% of trials required an anti-jump movement, touching the contralateral target location. While no group differences were shown on the DJRT, the DCD group were slower to complete reaching movements than the TDC group on AJRT; on the latter, the two DCD sub-groups were not shown to differ. Results confirm the presence of motor inhibition deficits in DCD which may not be dependent on the motor severity of the disorder.

## 1. Introduction

Developmental coordination disorder (DCD) is one of the most common neurodevelopmental disorders, with converging research showing a core deficit in predictive motor control (aka *internal modeling deficit*—IMD; Ruddock et al., 2016; Subara-Zukic et al., 2022), evident across effector systems including oculomotor (Katschmarsky et al., 2001), manual (Hyde and Wilson, 2011, 2013), and dynamic balance (Jelsma et al., 2016, 2020), as well as the integration of cognitive and motor control. This deficit manifests in slower response times, especially in response to unexpected changes in the environment, and more movement corrections given the child’s reliance on slower forms of feedback-based control (Wilson et al., 2013). However, it remains unclear whether this underlying issue in control depends on the severity of DCD: i.e., severe DCD (s-DCD) compared with mild-to-moderate DCD (m-DCD), and on the cognitive load of different movement tasks, issues that bear on our understanding of motor-cognitive coupling in everyday action (Ruddock et al., 2015, 2016).

Poor inhibitory control is commonly observed in DCD, which impacts the performance of tasks that require coupling of predictive motor and cognitive control. Inhibition is defined here as the ability to withhold or re-direct a motor response, often in the face of a prepotent stimulus (Ruddock et al., 2016). Predictive control (*viz* the ability to use forward estimates of limb position as a means of correcting an action rapidly in real time) is critical to motor coordination and skill development (McNamee and Wolpert, 2019). Ruddock and colleagues have shown that children with DCD are slower to make online corrections to target perturbations and less accurate than typically developing children (TDC) on both the double jump reaching task (DJRT) and anti-jump reaching task (AJRT). The latter task involves the ability to couple online predictive control and cognitive inhibition (Ruddock et al., 2016): the performer is required to monitor sudden jumps in target location (either to the left or right of fixation), but then inhibit a prepotent response and implement a reach movement to a contralateral location. Deficits in eye-limb coupling in DCD are also linked to poor inhibitory control and its integration with motor control (Michel et al., 2018). Sub-group differences have not been tested, however.

The downstream effects on motor performance are potentially quite profound for children with combined cognitive and motor control deficits. In longitudinal research, our group has shown that children with more severe motor coordination difficulties are likely to experience motor and cognitive issues in later development (Wilson et al., 2020). Children with persistent DCD had much poorer executive function than both typically developing children and those with remitting DCD. In short, the combination of persistent DCD and cognitive deficits is relatively common and predicts poorer developmental outcomes in later childhood.

An important theoretical and clinical question that remains unanswered is whether children with s-DCD (≤5th percentile on standardized tests of motor skills) show more profound difficulties in cognitive and motor control than those with m-DCD (between the 5th and 15th percentile). Combined deficits in predictive and inhibitory control may impede performance of visually-guided motor tasks, especially under time constraints and/or cognitive load (Ruddock et al., 2015). Children with s-DCD do perform worse on manual dexterity tasks which, by their nature, require a high degree of visuomotor integration (McQuillan et al., 2021). As well, in the case of motor imagery, an ability linked to internal modeling, Williams et al. (2008) showed that children with s-DCD had particular difficulty on a complex whole-body rotation task, unlike m-DCD who performed like controls. Such findings raise the question as to whether the severity of DCD constrains the ability to generate and utilize (predictive) internal models for action.

The goal of the study reported here was to compare the performance of large groups of children with s-DCD, m-DCD, and TDC on two versions of the visually-guided pointing task (DJRT and AJRT). Children with DCD were classified according to the level of motor impairment, measured by MABC-2: s-DCD (≤5th percentile) and m-DCD (TTS ≤16th percentile, but >5th). We predicted that the performance of children with DCD would be worse than TDC on key metrics of DJRT and AJRT performance, and, moreover, s-DCD would perform worse than m-DCD on each task.

## 2. Materials and methods

### 2.1. Participants

Eighty-two children with DCD (*M*age = 8.90 ± 1.63 years, age range 6–12 years, 37 girls/45 boys) were recruited, 29 with s-DCD (*M*age = 9.12 ± 1.56 years, age range 6–12 years 10 girls/19 boys) and 53 with m-DCD (*M*age = 8.78 ± 1.67 years, age range 6–12 years, 27 girls/26 boys), together with 201 TDC (*M*age = 9.20 ± 1.50 years, age range 6–12 years, 101 girls/100 boys), aged between 6 and 12 years, the latter forming part of a larger longitudinal study. As previous research has shown a large effect on group differences for both DJRT and AJRT (Hyde and Wilson, 2013; Ruddock et al., 2015), an *a priori* power analysis with G*Power 3.1 indicated that 28 participants in each group would achieve a desired power (1 − β) of 0.90, effect size d = 0.08, and an α level of 0.05 (Faul et al., 2007). We used the MABC-2 test battery to assess the level of motor competency in a large pool of children. In total, 201 TDC completed both the DJRT and AJRT. All children were recruited from four primary schools in the Olomouc region and surrounding communities in Moravia of the Czech Republic. All the schools were from urban areas/cities and the number of inhabitants in each city was over 25,000. Initially, the MABC-2 test battery was administered to all children within the age range of 6–12 years. Then, those children who performed under the 16th percentile were also assessed by teachers using the MABC-2 checklist. The study was approved by the Ethical Committee of the Faculty of Physical Culture, Palacký University Olomouc (FTK 46/2020), and participating schools. Informed consent was signed by the parents or legal guardians of the children, and oral assent was provided by each child before testing.

#### 2.1.1. Inclusion criteria

Children with DCD fulfilled four criteria of the Diagnostic and Statistical Manual of Mental Disorders, 5th edition (DSM-5; American Psychiatriac Association, 2013). As recommended by the European Academy of Childhood Disability (EACD; Blank et al., 2019), the Movement Assessment Battery for Children, 2nd edition (MABC–2 Test) (Henderson et al., 2007) was used to assess the level of children’s motor competence (criterion A). To evaluate the persistence of motor impairments in activity of daily living (criterion B), the MABC–2 checklist (Henderson et al., 2007) was completed by classroom teachers. The Checklist has excellent internal consistency, Cronbachʼs α > 0.92 (Schoemaker et al., 2012; Kita et al., 2016), good-to-excellent inter-rater reliability, ICC = 0.78–0.91 (Ramalho et al., 2013) and proven discriminant validity as a predictor of motor impairment (Schoemaker et al., 2012). The adapted Czech version of the Checklist is also sensitive to DCD and correlates significantly with the MABC-2 Test (*r*s = −0.31) (Banátová et al., 2022). School psychologists reviewed the medical and behavioral records of each child to assess criteria C and D. Twenty-one children were identified as having ADHD and excluded. Of these, 1 had comorbid autism spectrum disorder (ASD), and 9 a learning disorder. It should be acknowledged that even though this screening by school psychologists will help to exclude children with diagnosed conditions (e.g., ADHD, ASD), this will not identify children who are undiagnosed at that point as the rate of co-occurring conditions is high for children with DCD. Children who scored ≤16th percentile on the MABC–2 Test and MABC–2 checklist, and met DSM criteria C and D were classified as DCD. DCD subgroups were as follows: m-DCD if MABC-2 total score between the 6th to 15th percentile; s-DCD if ≤5th percentile. The TDC group all performed above the 20th percentile.

#### 2.1.2. Exclusion criteria

Children with an intellectual, physical, or sensory disability, or symptoms of a medical condition affecting movement were excluded.

### 2.2. Measurement instruments

The MABC-2 Test was administrated in the school gym by a group of examiners who underwent the training and they were certificated and experienced experts according to standardized guidelines in the Examiner’s Manual (Henderson et al., 2007). Standardized norms of the MABC-2 Test for the Czech population were used (Psotta, 2014). The MABC-2 Test has good validity and reliability across different age bands (Henderson et al., 2007; Schulz et al., 2011).

### 2.3. Apparatus and experimental task

The Double-Jump Reaching Task (DJRT) was used to assess online motor control. The DJRT paradigm was programmed using the VIRTOOLS Software Package (3DVIA, 2010), launched on a PC laptop, and displayed on a black Iiyama 43-in touchscreen monitor (Iiyama, Tokyo, Japan). The television was placed horizontally on a height-adjustable table in portrait orientation. All stimuli were displayed against a black background to reduce contrast interference.

The display consisted of a green “home base” circle and three yellow “target” circles, each of 25 mm in diameter. The home base circle was positioned in the middle bottom of the screen, 50 mm from the bottom edge of the display, and three yellow target circles at a distance of 40 mm above the home base, positioned at −20°, 0°, 20°. The home base was lit green when touched with the index finger and switched off at the point when the index finger was lifted from the surface. The child returned their index finger to the home base for each successive trial. To prevent the impact of anticipation, a random delay of 500–1,500 ms was used for target illumination. A successful trial occurred when the child touched the illuminated yellow target location within its circular boundary with the index finger; at the point of contact, the yellow light was extinguished, and an auditory tone emitted, indicating that the trial had ended. 80% of all trials were non-jump: the middle yellow target circle remained lit until touched by the index finger. The remaining 20% were jump trials: the yellow target location switched (or jumped) to either the left or right peripheral location at lift-off (or movement onset).

The AJRT task was administered in a separate block and was identical to the DJRT task, but with the exception that children were required to touch the contralateral target location on jump trials—referred to here as an anti-jump trial. 20% of all trials were anti-jump.

### 2.4. Procedure

The study was conducted at the university, in a quiet lab with normal fluorescent ceiling light and with no windows to avoid environmental distractions. Hand dominance was determined by observing the child’s preferred hand on manual dexterity items of the MABC-2 and self-report. Both typical DJRT and AJRT tasks were performed in two separate 10–15-min sessions, with the DJRT performed first. For the DJRT, each child was asked to stand behind the table adjusted to child waist and to hold their index finger on the green home base circle and then to reach and touch the center of one of the peripheral yellow target circles as quickly as possible, when illuminated. For AJRT task, each child was asked to reach and touch the (outlined) circle on the side opposite the lit circle (or stimulus)—defined as anti-jump trial. Each task was demonstrated prior to each experimental session to confirm that children understood the goal of each task and required actions for non-jump, jump, and anti-jump trials. Twenty practice trials were administered in each session. Each test session consisted of 80 trials divided into two blocks of 40 trials including 32 non-jump and 8 DJRT/AJRT, presented in a pseudo-random order (four each side) within 40 trials over the left- and right-side target locations.

### 2.5. Measures and statistical analysis

For each task, reaction time (RT) and movement time (MT) of each trial were recorded. MT was measured as the time interval between lift-off of the index finger of the dominant hand from the green “home base” to finger touch on the display. A successful trial was defined when the index finger touched within the circular boundary of the designated target location, both extinguishing the target and emitting an auditory signal which indicated successful completion of the trial (Ruddock et al., 2016). Unsuccessful trials (in which no response was initiated) or errors were excluded. A minimum of eight successfully completed jump/anti-jump trials per block was required (Ruddock et al., 2014). Average MT was calculated for jump, anti-jump, and non-jump trials. Next, outliers with values of ±1.5 *SD* from the average (Tukey, 1977) were removed from each group for both DJRT and AJRT. That is, if a child scored >1.5 *SD* in either the DJRT or AJRT, his/her data was removed for further statistical analysis. Consequently, for both DJRT and AJRT three children with s-DCD, three with m-DCD, and 14 TDC were excluded from further data analysis. For the DJRT, online control was measured by the movement time difference between *jump* and *no-jump* trials (MT^diff^), while for the AJRT, the coupling of online and inhibitory control was measured by the movement time difference between *jump* and *anti-jump* trials (AJMT^diff^; Ruddock et al., 2014).

Response errors were also recorded for each task. For both DJRT and AJRT, four error types were as follows: touch-down error (TDE), identified when the index finger touches the areas outside the yellow target spot; anticipatory error (AE) occurred when the index finger was lifted from the green “home base” circle before the yellow target was presented, or within 150 ms of stimulus display (Wilson et al., 1997); center touch error (CTE) occurred when the central target spot was touched instead of one of the peripheral target spots within the jump trial; and wrong-touch error (WTE) occurred when an incorrect (or cued target spot) was touched within anti-jump trial.

For each task, normality assumptions were tested using the Shapiro–Wilk test (*p* > 0.05). For each task, planned contrasts were conducted to compare MT^diff^ scores between groups, the first comparing TDC with a weighted average of m-DCD and s-DCD groups, and the second comparing the two DCD sub-groups. Error scores were compared between groups using Mann–Whitney *U* tests, conducted on AE, TDE, WTE, and CTE scores for DJRT and AJRT tasks. The magnitude of group differences was indexed using Cohen’s *d* and interpreted using standard benchmarks: low (*d* = 0.2), medium (*d* = 0.5), and large (*d* = 0.8) effect (Cohen, 1988). To estimate the effect sizes in the non-parametric Mann–Whitney *U* test, the *r* effect size was calculated by dividing the obtained *z* score by the square root of the sample size number (Fritz et al., 2012). These *r* values were then transformed into the equivalent of Cohen’s *d* values using the formula *d* = [(√h)*r]/[√1 − *r*^2^] where h = [(*n*1 + *n*2 − 2)/*n*1] + [(*n*1 + *n*2 – 2)/*n*2] (Cohen, 1988; Borenstein et al., 2009; Lenhard and Lenhard, 2016). A Spearman’s rho correlation test was used to estimate the correlations between MT^diff^, AJMT^diff^, and AE, TDE, WTE, and CTE errors, respectively.

## 3. Results

### 3.1. Age group

An independent *t*-test showed that there was no significant difference in the mean age of DCD and TDC groups, *t*(280) = 1.496, *p* = 0.136 as well as between m-DCD and s-DCD groups, *t*(80) = −0.915, *p* = 0.363.

### 3.2. MT^diff^ and MT

The results of planned contrasts for MT^diff^ and MT in each group are presented in Table 1.

**Table 1:** Descriptive statistics for the double jump reaching task (DJRT) and anti-jump reaching task (AJRT): movement time (MT) and movement time difference (MT^diff^), expressed for each group [typically-developing children (TDC) versus developmental coordination disorder (DCD)], as a function of motor severity [moderate DCD (m-DCD) versus severe-DCD (s-DCD)], and age (younger: 6–8 years; older: 9–12 years).

DJRT							
	Groups	*N*	Mean	Std. deviation	*t*	*p*	*d*
MT^diff^	TDC	201	220.20	79.66	−0.435	0.664	0.08
	DCD	82	227.06	97.58			
	m-DCD	53	231.51	92.25	0.638	0.524	0.12
	s-DCD	29	218.93	107.87			
MT^diff^ (6–8 years old)	TDC	68	236.50	95.2	−0.237	0.813	0.15
	DCD	37	251.86	105.49			
	m-DCD	27	263.85	89.89	0.214	0.228	0.42
	s-DCD	10	219.50	139.89			
MT^diff^ (9–12 years old)	TDC	133	211.87	69.33	−0.279	0.781	0.07
	DCD	45	206.67	86.5			
	m-DCD	26	197.92	83.62	−0.927	0.355	0.23
	s-DCD	19	218.63	91.19			
MT (no-jump)	TDC	201	623.01	160.01	1.767	0.078	0.22
	DCD	82	660.60	188.43			
	m-DCD	53	653.81	172.15	−0.492	0.623	0.10
	s-DCD	29	673.00	217.77			
MT (jump)	TDC	201	843.21	150.31	−2.167	0.031	0.28
	DCD	82	887.66	164.66			
	m-DCD	53	885.32	155.15	−0.185	0.854	0.04
	s-DCD	29	891.93	183.69			

AJRT							
	Groups	*N*	Mean	Std. deviation	*t*	*p*	*d*
MT^diff^	TDC	201	532.92	164.11	−2.819	0.005	0.37
	DCD	82	596.54	187.31			
	m-DCD	53	592.25	185.27	−0.306	0.759	0.06
	s-DCD	29	604.38	194.03			
MT^diff^ (6–8 years old)	TDC	68	634.63	156.81	−2.81	0.006	0.44
	DCD	37	708.70	183.01			
	m-DCD	27	675.81	180.7	−2.004	0.048	0.79
	s-DCD	10	797.50	166.27			
MT^diff^ (9–12 years old)	TDC	133	480.92	142.38	−0.947	0.345	0.16
	DCD	45	504.31	133.81			
	m-DCD	26	505.46	148.54	0.064	0.949	0.02
	s-DCD	19	502.74	114.52			
MT (no-jump)	TDC	201	711.16	184.32	−1.616	0.107	0.81
	DCD	82	757.20	203.01			
	m-DCD	53	768.00	200.31	0.696	0.487	0.15
	s-DCD	29	737.45	209.96			
MT (jump)	TDC	201	1224.08	229.12	−3.327	0.001	0.54
	DCD	82	1353.73	255.99			
	m-DCD	53	1360.25	319.03	0.336	0.737	0.06
	s-DCD	29	1341.83	241.95			

### 3.3. Errors

**Figure 1:**
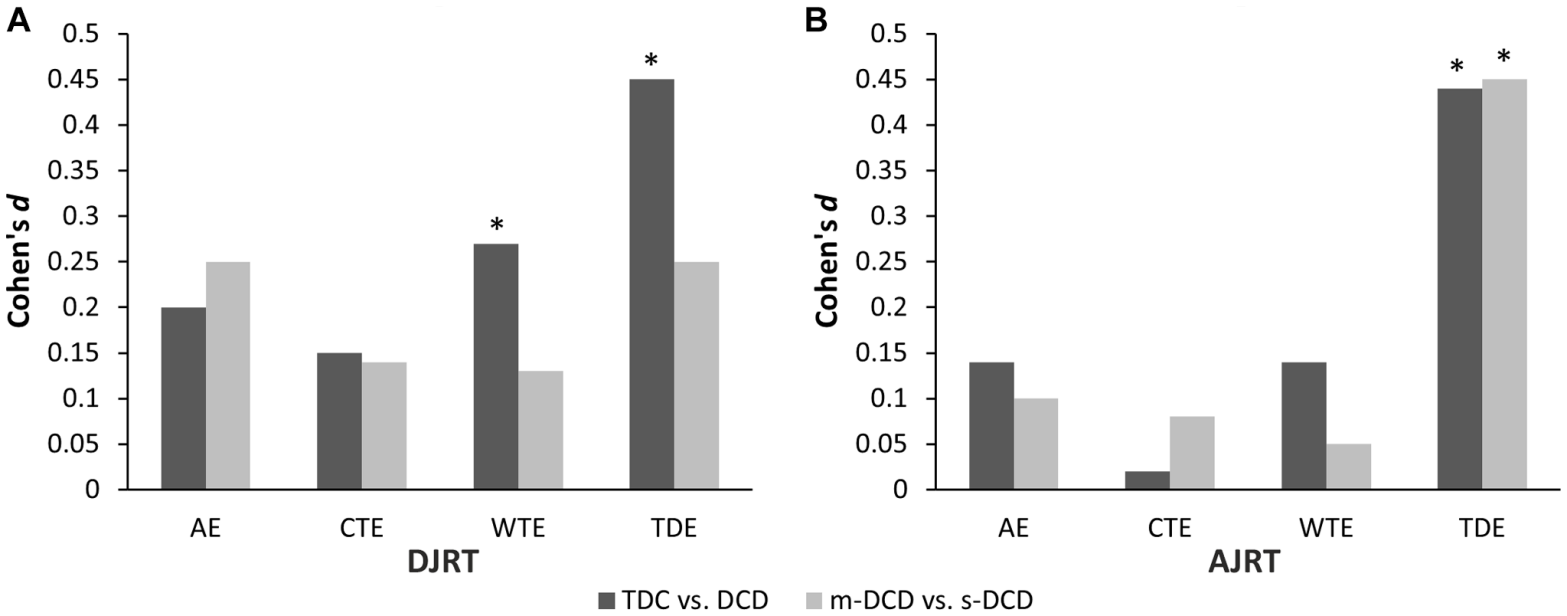
Effect size estimates Cohen’s *d* for error differences between typically-developing children (TDC) versus developmental coordination disorder (DCD), and moderate DCD (m-DCD) versus. Severe DCD (s-DCD) in double-jump reaching task (DJRT) **(A)** and in anti-jump reaching task (AJRT) **(B)**.

For both DJRT and AJRT, the results of the non-parametric Mann–Whitney *U* tests on AE, CTE, WTE, and TDE errors and effect sizes comparing TDC and DCD are presented in Table 2 and Figure 1A, and between m-DCD and s-DCD are presented in Table 2 and Figure 1B, respectively.

**Table 2:** Means, median, standard deviations, minimum, maximum, and comparison of error scores on double jump reaching task (DJRT) and anti-jump reaching task (AJRT) across the groups: typically-developing children (TDC) versus developmental coordination disorder (DCD), and moderate DCD (m-DCD) versus severe-DCD (s-DCD).

DJRT										
	Groups	Mean	Median	Std. deviation	Minimum	Maximum	*U*	*z*	*p*	*d*
AE	TDC	2.30	1	3.75	0	33	7192.5	−1.718	0.086	0.20
	DCD	2.54	2	3.19	0	23				
	m-DCD	2.11	1	2.16	0	10	650.5	−1.166	0.244	0.25
	s-DCD	3.31	2	4.46	0	23				
CTE	TDC	0.21	0	0.54	0	3	7456.0	−1.894	0.058	0.22
	DCD	0.39	0	0.84	0	4				
	m-DCD	0.38	0	0.90	0	4	699.0	−0.899	0.369	0.19
	s-DCD	0.41	0	0.73	0	3				
WTE	TDC	0.23	0	0.62	0	4	6814.0	−3.206	<0.01	0.38
	DCD	0.57	0	1.04	0	5				
	m-DCD	0.53	0	1.04	0	5	704.5	−0.747	0.455	0.16
	s-DCD	0.66	0	1.04	0	4				
TDE	TDC	3.54	3	3.04	0	16	5925.5	−3.737	<0.001	0.45
	DCD	0.57	0	1.04	0	5				
	m-DCD	0.53	0	1.04	0	5	651.5	−1.142	0.254	0.25
	s-DCD	0.66	0	1.04	0	4				

AJRT										
	Groups	Mean	Median	Std. deviation	Minimum	Maximum	*U*	*z*	*p*	*d*
AE	TDC	2.31	2	2.41	0	15	7488.5	−1.227	0.220	0.14
	DCD	2.99	2	3.40	0	17				
	m-DCD	2.92	1	3.51	0	17	718.5	−0.496	0.620	0.10
	s-DCD	3.10	2	3.25	0	15				
CTE	TDC	0.06	0	0.27	0	2	8129.5	−0.492	0.623	0.05
	DCD	0.04	0	0.18	0	1				
	m-DCD	0.02	0	0.13	0	1	730.0	−1.148	0.251	0.25
	s-DCD	0.07	0	0.25	0	1				
WTE	TDC	0.45	0	0.76	0	4	7471.0	−1.468	0.142	0.17
	DCD	0.59	0	0.86	0	4				
	m-DCD	0.58	0	0.81	0	3	743.0	−0.282	0.778	0.06
	s-DCD	0.59	0	0.94	0	4				
TDE	TDC	2.63	2	2.31	0	13	5945.0	−3.715	<0.001	0.45
	DCD	4.07	3	3.42	0	21				
	m-DCD	3.28	3	2.08	0	8	560.5	−2.041	0.041	0.46
	s-DCD	5.52	4	4.73	0	21				

AE, anticipatory error; CTE, central touch error; WTE, wrong-touch error; TDE, touch-down error.

### 3.4. Correlations between MT^diff^ and errors

Spearman correlations between MT^diff^ and Error scores on each task (DJRT and AJRT) are presented for each participant group in Table 3.

**Table 3:** Spearman correlations between movement time difference (MT^diff^) and anticipatory error (AE), central touch error (CTE), wrong-touch error (WTE), touch-down error (TDE) on each task [double-jump reaching task (DJRT) and anti-jump reaching task (AJRT)], presented as a function of group [typically developing children (TDC) versus developmental coordination disorder (DCD), and moderate-DCD (m-DCD) versus severe-DCD (s-DCD)].

DJRT						
			AE	CTE	WTE	TDE
TDC (*n* = 201)	MT^diff^	*r*s	0.00	0.24	0.05	0.15
		*p*	0.953	<0.001	0.443	0.030
DCD (*n* = 82)	MT^diff^	*r*s	−0.17	0.26	−0.04	0.35
		*p*	0.115	0.018	0.693	<0.001
m-DCD (*n* = 54)	MT^diff^	*r*s	−0.08	0.40	−0.13	0.31
		*p*	0.565	0.003	0.351	0.023
s-DCD (*n* = 28)	MT^diff^	*r*s	−0.34	0.01	0.11	0.40
		*p*	0.063	0.941	0.549	0.031

AJRT						
			AE	CTE	WTE	TDE
TDC (*n* = 201)	MT^diff^	*r*s	0.94	0.15	0.22	0.29
		*p*	0.183	0.028	0.002	<0.001
DCD (*n* = 82)	MT^diff^	*r*s	0.243	0.144	0.266	0.517
		*p*	0.028	0.197	0.016	<0.001
m-DCD (*n* = 54)	MT^diff^	*r*s	0.17	0.08	0.30	0.46
		*p*	0.202	0.561	0.024	<0.001
s-DCD (*n* = 28)	MT^diff^	*r*s	0.38	0.19	0.17	0.64
		*p*	0.041	0.310	0.378	<0.001

## 4. Discussion

The primary goal of our study was to compare the ability of children with m-DCD, s-DCD, and TDC on two versions of a visual perturbation task: the DJRT that requires (automatic) online adjustments to a new target location, and the AJRT that requires coupling of rapid online control and response inhibition. Performance of the DJRT was shown to be comparable between TDC, m-DCD and s-DCD groups, while for the AJRT, the DCD group at large performed worse than TDC, while no difference was shown between m-DCD and s-DCD. In addition, correlational data suggested a link between MT^diff^ scores and TDEs across all groups. These findings have important implications for our understanding of cognitive-motor coupling in DCD, discussed below.

Contrary to previous studies (Hyde and Wilson, 2013; Ruddock et al., 2015), our results failed to show performance differences between DCD and TDC on the DJRT, a task that requires rapid online corrections based on a forward estimate of limb trajectory. The earlier Australian studies showed significantly larger MT^diff^ scores for DCD groups, as well as longer response times to change reach trajectory on jump trials. More specifically, using a target distance of 30 cm, Hyde and Wilson (2011) reported mean scores of 338 ms for DCD compared with 260 ms for TD. As well, in a comparison of DCD, age-matched control (AMC), and younger controls (YC), Hyde and Wilson (2013) showed a similar performance pattern between DCD and YC, suggesting a developmental immaturity in rapid online control: MT^diff^ for these two groups was 344 and 388 ms, respectively, compared with 275 ms for AMC. In the current study, corresponding MT^diff^ values were slightly faster overall: 227 ms for DCD and 220 for controls. It is possible that the absence of a group effect here compared with earlier studies is due to differences in participant demographics and contextual factors (i.e., regional vs. large cities), which influence the physical activity levels of the respective DCD samples, discussed below.

For the AJRT, results confirmed deficits in DCD when coupling rapid online (motor) control with inhibitory control (Ruddock et al., 2015). The earlier study by Ruddock et al. (2015) compared DCD and TD groups at three different ages: younger (6–7 years), mid-aged (8–9 years), and older (10–12 years). Younger and mid-aged children with DCD were disadvantaged on anti-jump trials relative to their age-matched controls; e.g., for younger children, AJMT^diff^ scores were 499 ms versus 352 ms, respectively, and for mid-aged children, 359 ms versus 248 ms. For older children, the difference was not significant between motor groups: 207 ms versus 210 ms. In the current study, AJMT^diff^ was higher for the total DCD group (597 ms) than TD (533 ms). Taken together, as cognitive control develops steadily over the childhood period and beyond (Friedman et al., 2009; Luna, 2009), it is likely that the reduced level of performance on the AJRT in children with DCD may reflect delayed development of cognitive control and its coupling to feedforward/predictive motor control. This hypothesis is supported by data showing that group differences are largely confined to younger cohorts, consistent with our study reported here and earlier work (Hyde and Wilson, 2011; Ruddock et al., 2015, 2016).

This deficit in cognitive-motor coupling may explain the performance difficulty that these children display on more complex motor tasks that present cognitive planning, sequencing, or dual-task components (Wilson et al., 2013). This is shown, for example, by motor planning difficulties in DCD when the complexity of the task increases (Krajenbrink et al., 2020, 2021). Immaturity in reciprocal connectivity between frontal and posterior control systems may impair the integration of cognitive control with real-time adjustments to movement trajectory (Ruddock et al., 2015). While deficits in cognitive inhibition appear less pronounced with age over childhood (Ruddock et al., 2015), the combination of motor skill and executive function difficulties in children is likely to be a risk factor for persisting DCD (Wilson et al., 2020). Overall, the difficulty in inhibitory control and its integration with online motor control is a fundamental issue in children with DCD when performing speeded pointing movements.

Comparable performance of m-DCD and s-DCD sub-groups on the DJRT is somewhat at odds with sub-group differences observed for manual dexterity (e.g., pegboard placement, threading and drawing items of the MABC-2; McQuillan et al., 2021). Perturbation trials on both the DJRT and AJRT require rapid responses performed under open-loop control, while simple manual dexterity tasks are more closed-loop (or feedback dependent). Children with DCD “live on feedback” (Clark, personal communication, 2011) — a mode of control that is not optimal for tasks that demand rapid online corrections. Put another way, because the planning process is not complete before the start of a task (aka IMD), children with DCD rely more on feedback over the course of movement, and therefore have a slower, more iterative mode of motor control, adjusting their movements in successive steps. This raises the intriguing hypothesis that the nature of the motor task (and the attendant demands it imposes on open-loop motor control) will determine whether performance difficulties are generalized across DCD sub-groups. Put another way, in the case of anti-jump reaching, demands on cognitive-motor coupling were complex enough to influence the performance of children with DCD, regardless of their motor severity.

More frequent touch errors in DCD (i.e., TDEs on both tasks and CTEs on the AJRT) suggest a generalized difficulty with endpoint and/or trajectory control, seen also in a range of other target-directed pointing and reaching tasks (Mandich et al., 2002; Wilmut et al., 2007; Hyde and Wilson, 2013). At the sub-group level, more TDEs in s-DCD relative to m-DCD on the AJRT suggests that endpoint control is more compromised in s-DCD under an inhibitory load. The absence of any group difference on AEs or WTEs suggests that children were well-oriented to task instructions and performed consistently in reference to task goals. Future research should examine performance accuracy on other measures of response inhibition and cancellation to determine the effect of different levels of response expectancy on performance.

Some distinctions in demographics and context between the Czech Republic and Australian study, may explain some of the discrepancies between our current and earlier studies. There has been accumulating evidence that residential context (or physical environment) is one of the main determinants of children’s physical activity (Kimbro et al., 2011; Sharp et al., 2015), correlated also with physical fitness and motor coordination (Amador-Ruiz et al., 2018; Gallotta et al., 2022). In the earlier Australian studies, children were drawn from the large city of greater Melbourne, mainly its densely populated inner suburbs (Hyde and Wilson, 2013; Ruddock et al., 2015). Although we did not measure physical activity specifically, children in the Czech sample – recruited from the regional city of Olomouc and surrounding communities in Moravia – were more likely to engage in outdoor recreational activities than those in the Australian (urban) sample. One hypothesis worth testing is whether higher levels of physical activity in children meeting criteria for DCD may inoculate them against more severe functional impairments.

Our findings have some important implications for practitioners who work with DCD, most notably the importance of considering cognitive load when designing training tasks. Such tasks should be scaled in difficulty not only in motoric terms but also cognitive. In the case of dual-tasks, for example, dual-task interference tends to be higher for more complex primary motor tasks compared with simple tasks, and the experience of fatigue is much higher in DCD (Krajenbrink et al., 2023). Cognitive-motor dual-tasks may be used as effective training tools, much like that demonstrated in the neurorehabilitation field (Li et al., 2020; Pereira Oliva et al., 2020; Johansson et al., 2023).

In sum, our study suggests that deficits in cognitive-motor coupling are prominent in children with DCD, regardless of the severity of motor skill impairment. Specifically, difficulties integrating cognitive control when performing a speeded task presents both a speed and accuracy cost. The likely downstream effect is performance difficulty on complex tasks that involve both visuomotor coordination and cognitive processing, e.g., dual-tasks like navigating on foot while solving a cognitive task, or sequential motor tasks that require cognitive problem solving. Future research should consider involving older children and adults, careful screening of comorbid conditions that may impact inhibition, prior levels of physical activity (i.e., motor experience), and consideration of IQ and its relationship to executive function and visio-spatial constructional ability (Vaivre-Douret et al., 2020).

## Data availability statement

The datasets presented in this article are not readily available because of Human Subjects’ protections. Requests to access the datasets should be directed to RA, reza.abdollahipour@upol.cz.

## Ethics statement

The studies involving humans were approved by the Ethical Committee of the Faculty of Physical Culture, Palacký University Olomouc (FTK 46/2020), and participating schools. The studies were conducted in accordance with the local legislation and institutional requirements. Written informed consent for participation in this study was provided by the participants’ legal guardians/next of kin.

## Author contributions

RA, LV, KB, LB, TK, ZS, and PW: conceptualization, methodology, project administration, and visualization. RA, LV, and PW: formal analysis. ZS and PW: supervision. BS: interpretation of results. RA, LV, KB, LB, TK, ZS, BS, and PW: data curation, investigation, drafting, and review and editing. All authors contributed to the article and approved the submitted version.
